# A Multi-Analyte Assay for the Non-Invasive Detection of Bladder Cancer

**DOI:** 10.1371/journal.pone.0047469

**Published:** 2012-10-19

**Authors:** Steve Goodison, Myron Chang, Yunfeng Dai, Virginia Urquidi, Charles J. Rosser

**Affiliations:** 1 Cancer Research Institute, M.D. Anderson Cancer Center Orlando, Orlando, Florida, United States of America; 2 Department of Biostatistics, The University of Florida, Gainesville, Florida, United States of America; 3 Section of Urologic Oncology, MD Anderson Cancer Center Orlando, Orlando, Florida, United States of America; 4 Nonagen Bioscience Corp, Orlando, Florida, United States of America; Florida International University, United States of America

## Abstract

Accurate urinary assays for bladder cancer (BCa) detection would benefit both patients and healthcare systems. Through genomic and proteomic profiling of urine components, we have previously identified a panel of biomarkers that can outperform current urine-based biomarkers for the non-invasive detection of BCa. Herein, we report the diagnostic utility of various multivariate combinations of these biomarkers. We performed a case-controlled validation study in which voided urines from 127 patients (64 tumor bearing subjects) were analyzed. The urinary concentrations of 14 biomarkers (IL-8, MMP-9, MMP-10, SDC1, CCL18, PAI-1, CD44, VEGF, ANG, CA9, A1AT, OPN, PTX3, and APOE) were assessed by enzyme-linked immunosorbent assay (ELISA). Diagnostic performance of each biomarker and multivariate models were compared using receiver operating characteristic curves and the chi-square test. An 8-biomarker model achieved the most accurate BCa diagnosis (sensitivity 92%, specificity 97%), but a combination of 3 of the 8 biomarkers (IL-8, VEGF, and APOE) was also highly accurate (sensitivity 90%, specificity 97%). For comparison, the commercial BTA-Trak ELISA test achieved a sensitivity of 79% and a specificity of 83%, and voided urine cytology detected only 33% of BCa cases in the same cohort. These datashow that a multivariate urine-based assay can markedly improve the accuracy of non-invasive BCa detection. Further validation studies are under way to investigate the clinical utility of this panel of biomarkers for BCa diagnosis and disease monitoring.

## Introduction

The non-invasive detection and monitoring of bladder cancer (BCa) remains a challenge. Voided urinary cytology (VUC) remains the most established urine-based assay for this purpose, but while VUC has a specificity of >93%, the assay suffers from low sensitivity (25–40%) and observer-dependent variability [1].Commercial tests measuring nuclear matrix protein (NMP-22) and bladder tumor antigen (BTA) have emerged as diagnostic urinary protein tests for BCa, but these single-marker assays lack the specificity of VUC [2], [3]. The concept that the presence or absence of one molecular marker will aid clinical evaluation has not proved to be the case. This is not surprising when one considers the variation between individuals, the cross-talk between molecular pathways, and the heterogeneity of solid tumors.

The advent of high-throughput techniques has enabled an evolution from single-marker research to a more global assessment strategy, and we have used these to identify promising novel biomarkers of BCa. Using genomics [4] and proteomics [5] approaches to profile the soluble and cellular components of voided urine, we have identified a panel of biomarkers that show promise for development into accurate assays for non-invasive BCa detection. We have performed a series of validation studies [6]–[9] to evaluate the potential clinical utility of a number of these biomarkers. In this study, we combined data on 14 of our candidate biomarkers in a cohort of 127 subjects in order to derive an accurate and robust multivariate assay for the non-invasive detection of BCa.

## Patients and Methods

### Specimen and Data Collection

Under Institutional Review Board approval and informed consent (IRB# 560–2006), voided urine samples, and associated clinical information were prospectively collected into a genitourinary tissue bank. Prior to any type of therapeutic intervention, ∼100 mL of voided urine was obtained from each subject. Fifty milliliters of urine was used for clinical laboratory analyses per standard procedures. The remaining urine aliquot was assigned a unique identifying number before immediate laboratory processing. Each urine sample was centrifuged at 600×*g* 4°C for 5 min. The supernatant was decanted and aliquoted, while the urinary pellet was snap frozen. Both the supernatant and pellet were stored at −80°C prior to analysis. Protein content of aliquots was measured using a Pierce 660-nm Protein Assay Kit (Thermo Fisher Scientific Inc., Waltham, MA, USA) and a NanoDrop spectrophotometer (ND-1000, ThermoScientific, Wilmington, DE, USA). The tissue bank was queried for suitable specimens for analysis, which included non-consecutive samples from 127 subjects. The study cohort consisted of 63 individuals without previous history of urothelia cell carcinoma, gross hematuria, active urinary tract infection or urolithiasis (65% with voiding symptoms and 35% with microscopic hematuria) and 64 individuals with newly diagnosed urothelial cell carcinoma. Specimens from patients with known renal disease or documented renal insufficiency were not included. According to the International Consensus Panel on Bladder Tumor Markers [10], this cohort served as a phase II (validation study). Data is reported using the STARD criteria [11]. All subjects were evaluated in the outpatient Urology clinic. Urinalysis and VUC were performed on all subjects. All subjects underwent office cystoscopy and axial imaging of the abdomen and pelvis.In the cancer group, post-operative histological confirmation of urothelial cell carcinoma, including grade and stage, was recorded. Pertinent information on clinical presentation, staging, histologic grading [12], [13] and outcome are presented in **Table 1**. Median follow-up of our control and cancer cohorts was 11.5 and 12.0 months, respectively.

**Table 1:** Demographic and clinicopathologic characteristics of the study cohort.

	Non-cancer (%)N = 63	Cancer (%)N = 64
Median Age (range, y)	60 (30–81)	69.5 (22–90)
Male : Female ratio	55∶8	55∶9
**Race**		
White	41 (65)	58 (91)
African American	8 (13)	0 (0)
Other	14 (22)	6 (9)
Gross hematuria	0 (0)	47 (73)
Suspicious/positive cytology	0 (0)	21 (33)
Median follow-up (months)	11.5	12.0
**Clinical stage**		
Tis∧	n/a	6 (9)
Ta	n/a	15 (23)
T1	n/a	9 (14)
T2	n/a	31 (48)
T3	n/a	4 (6)
T4	n/a	2 (3)
N+ ∼	n/a	3 (5)
**Grade**		
Low	n/a	9 (14)
High	n/a	55 (86)
**Median tumor size (cm)**	n/a	4.5

∧, 4 subjects with concomitant cis had T1 (n = 2) and T2 (n = 2) disease.

∼, Subjects with T2 (n = 1), T3 ( = 1) and T4 (n = 1) disease and node positive.

### Enzyme-linked Immunosorbent Assays for 14 Urinary Biomarkers and Hemoglobin

Levels of human interleukin 8 (IL-8, Cat # ab46032 Abcam, Cambridge, MA, USA), Matrix metalloproteinase 9 (MMP-9, Cat# DMP900 R&D Systems Inc., Minneapolis, MN, USA), Syndecan (SDC-1, Cat# ab46507 Abcam, Cambridge, MA, USA), chemokine (C-C motif) ligand 18 (CCL18, Cat # ab100620 Abcam, Cambridge, MA, USA), Plasminogen Activator Inhibitor 1 (PAI-1, Cat# EA-0207 Signosis Inc., Sunnyvale, CA, USA), CD44 (Cat # ab 45912Abcam, Cambridge, MA, USA), Vascular endothelial growth factor(VEGF, Cat # 100663 Abcam, Cambridge, MA, USA), Angiogenin (ANG,Cat# CK400 CellSciences, Canton, MA, USA), Carbonic anhydrase 9 (CA9, Cat# DCA900 R&D Systems Inc., Minneapolis, MN, USA), Alpha 1-Antitrypsin (A1AT, Cat # ab108799 Abcam, Cambridge, MA, USA), Osteopontin (OPN, Cat# DOST00 R&D Systems, Inc., Minneapolis, MN, USA), Pentraxin 3 (PTX3, Cat# DPTX30 R&D Systems, Inc., Minneapolis, MN, USA) and human Apolipoprotein E (APOE, Cat # KA 1031 Abnova, Walnut, CA, USA) were monitored in urine samples using commercial enzyme-linked immunosorbent assays (ELISA). A commercial assay (BTA-Trak© Ca# 662150 Polymedco Inc. Cortlandt Manor, NY, USA) for BCa detection, and that is available in ELISA format, was also monitored in each urine sample. In addition, a commercially available ELISA assay was used to measure levels of urinary hemoglobin (Cat#E88–135 Bethyl Laboratories Inc., Montgomery, TX, USA).ELISA assays were conducted according to the manufacturer’s instructions. Calibration curves were prepared using purified standards for each protein assessed. Curve fitting was accomplished by either linear or four-parameter logistic regression following manufacturer’s instructions. Laboratory personnel were blinded to final diagnosis.

Due to the unavoidable variability of voided urine with respect to total volume and time within the bladder, each biomarker was normalized to urinary creatinine as previously described [5]–[9]. The concentration of urinary creatinine was measured using a commercially available enzymatic assay (Cat# KGE005 R&D Systems Inc., Minneapolis, MN, USA) according to the manufacturer’s instructions. Creatinineconcentrations of unknown samples were calculated by comparison to a standard curve.

### Data Analysis

We investigated the diagnostic performance of 14 urinary biomarkers for BCa detection, both individually and in all combinations. We used Wilcoxon rank sum tests to determine the association between each biomarker and BCa. For combinatorial analyses, the goals were to identify the most accurate multivariate models overall, but also to define accurate models using a minimum number of necessary biomarkers. For model selection, we applied the logistic regression procedure with BCa status (Yes vs. No) as the response variable and the biomarkers as predictive variables.We used the all-subset method to evaluate the predictive value of each possible subset of biomarkers. The Bayesian information criterion (BIC) was used to compare models [14]. The BIC, a widely used criterion in model selection, balances the model likelihood and the number of biomarkers included in the model.Automated variable selection methods, such as step-wise selection, may produce unstable models [15]. As suggested by Austin and Tu [15], we used the Bootstrap method (using 1000 Bootstrap samples) to select the most efficient and stable model to predict the presence of BCa. For Bootstrap sampling, we used the stratification technique: Bootstrap samples were taken from subjects with BCa and without BCa separately, and the two samples were merged together to form an overall Bootstrap sample. The stratified sampling technique ensures that the numbers of subjects with BCa and without BCa in a Bootstrap sample were the same as in the original dataset. For each Bootstrap sample, we evaluated the prediction value of each candidate subset of biomarkers usinga logistic regression model, and all candidate subsets of biomarkers were ranked from the top to the bottom by BIC. Then the subset of biomarkers that was ranked at the top most frequently among the 1000 Bootstrap samples was selected as the best combination of biomarkers for the prediction of BCa.

After the predictive model was selected, we generated nonparametric receiver operating characteristic (ROC) curves that plotted the value for sensitivity against the false-positive rate (1-specificity). The relative ability of the combination of selected biomarkers to indicate BCa was estimated by calculating the area under the ROC curves (AUC), with a higher AUC indicating a stronger predictor. We compared AUCs by chi-square test. With each individual biomarker, we estimated the sensitivity and specificity of each biomarker at the optimal cutoff value defined by the Youden index [16], i.e. the cutoff value that maximizes the sum of the sensitivity and the specificity. With combinations of the biomarkers, we first used the Bootstrap method to identify the most accurate model using a minimum number of necessary biomarkers as described in Data Analysis section. We found that the combination of IL-8, VEGF, and APOE was the best subset of the 8 biomarkers in prediction of BCa. We then performed the logistic regression analysis with BCa status (yes vs. no) as the response variable and with IL-8, VEGF, and APOE as the predictive variables. We used the regression coefficients estimated in the logistic regression as coefficients to form a linear combination of IL-8, VEGF, and APOE, and used the linear combination to predict BCa. The sensitivity and specificity of combination of IL-8, VEGF, and APOE was determined at the optimal cutoff value for the linear combination, i.e. the cutoff value that maximizes the sum of the sensitivity and the specificity. Statistical significance in this study was set at *p*<0.05 and all reported *p* values were 2-sided. All analyses were performed using SAS software version 9.3.

## Results

Demographic, clinical and pathologic characteristics of the 127 subjects who comprised our study group are illustrated in **Table 1**. Five subjects (3 in control group, 2 in cancer group) with missing biomarker data were excluded from final analysis.No subjects in the control cohort had an abnormal cystoscopy or axial imaging. Furthermore in follow-up, none of the control subjects were noted to develop BCa or even gross hematuria. In the cancer cohort, 41% of subjects had non-muscle invasive disease and 19% of subjects had low-grade disease. The median tumor size was 4.5 cm. In line with our previous experience [4], voided urinary cytology (VUC) in the cohort achieved 98% specificity, but only 33% sensitivity.

The mean and median levels of the 14 biomarkers in urine are presented in **Table 2** **.** The levels of 10 of the biomarkers were significantly elevated in subjects with BCa, relative to subjects without BCa. CD44 and OPN were significantly, but negatively, associated with BCa, and SDC1 and PTX3 were not significantly associated with BCa. Given the fact that the majority of patients present at the urology clinic with hematuria, one has to consider whether specific biomarker assays are detecting a tumor antigen or a serum protein introduced into the test sample via bleeding. To control for this possibility, we quantitated the hemoglobin level in all samples and evaluated the correlation of all 14 biomarkers with this measure of hematuria. This analysis revealed that levels of CCL18 and A1AT had a high correlation to urinary hemoglobin (Spearman’s correlation coefficient >0.8), and so these were not included in the multivariate models described below.

**Table 2:** Mean and median urinary levels of 14 biomarkers assessed by ELISA.

Biomarker	Cancer		Normal		P-value*
	Mean ± StdDev	Median [Min, Max]	Mean ± StdDev	Median [Min, Max]	
IL8 (pg/ml)	1368.08±3546.65	128.43 [0, 17140.16]	5.18±21.32	0 [0, 134.33]	<0.0001
MMP9 (ng/ml)	47.42±143.23	0.9 [0, 1002.6]	0.34±1.85	0 [0, 14.25]	<0.0001
SDC1 (ng/ml)	56.59±75.57	33.33 [0, 335.18]	48.40±42.45	38.62 [0, 199.55]	0.487
CCL18 (pg/ml)	654.89±1640.48	57.26 [0, 9523.04]	4.48±9.73	0 [0, 37.69]	<0.0001
PA1 (ng/ml)	7.18±21.04	0.25 [0.25, 125.26]	0.28±0.08	0.25 [0.25, 0.64]	<0.0001
CD44 (ng/ml)	53.97±56.05	28.73 [16.67, 344.04]	117.71±104.76	87.38 [16.08, 616.3]	<0.0001
VEGF (pg/ml)	873.69±1757.89	339.98 [0, 9841.4]	69.34±198.38	0 [0, 904.76]	<0.0001
ANG (pg/ml)	1530.34±3252.44	436.96 [3.28, 17944]	124.70±163.90	42.78 [20.48, 696.18]	<0.0001
CA9 (pg/ml)	115.11±536.60	10.36 [0, 4132.9]	3.84±5.65	0 [0, 28.28]	<0.0001
A1AT (ng/ml)	3487.06±10839.43	1227.15 [11.18, 83296]	158.16±397.63	39.48 [5.93, 2448.85]	<0.0001
OPN (ng/ml)	553.25±793.13	240.84 [0.27, 3926]	1643.61±2012.57	979.45 [0, 11120]	<0.0001
PTX3 (ng/ml)	1.17±1.67	0.92 [0, 12.5]	0.90±0.89	0.64 [0, 2.63]	0.267
APOE (pg/ml)	180.0±330.0	70.0 [10.0, 1780]	30.0±30.0	20.0 [0, 110]	<0.0001
MMP10 (pg/ml)	101.74±157.45	75.31 [0, 1239.84]	62.21±50.56	45.86 [0, 226.12]	0.008

* Two-sided p-values obtained from Wilcoxon rank sum tests.

**Figure 1:**
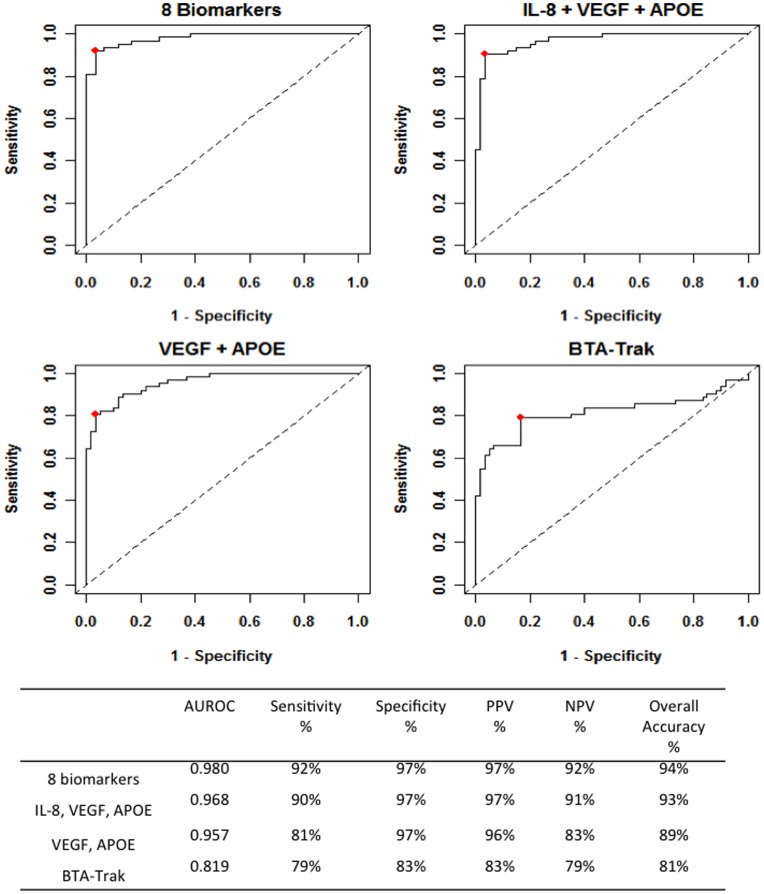
Diagnostic performance of urinary biomarker combinations. ROC curves were plotted to compare performance characteristics of the total 8-biomarker combination, a 3-biomarker combination (IL-8, VEGF, APOE), a 2-biomarker combination (VEGF, APOE), and the BTA-Trak test. Based on the area under the ROC curve (AUROC),Youden Index cutoff values that maximized the sum of sensitivity and specificity were determined for each biomarker (crossed square on curve). Table provides performance values for each combination. PPV, positive predictive value. NPV, negative predictive value.

**Figure 1** illustrates the diagnostic performance of various combinations of biomarkers. When all 8 positively associated BCa biomarkers were combined, the overall diagnostic accuracy was 94%, with a sensitivity of 92% and a specificity of 97%. Logistic regression and a 1000 Bootstrap sample strategywere performed to determine optimal diagnostic biomarker combinations, i.e. high accuracy with as few biomarkers as possible.For each Bootstrap sample, candidate subsets were ranked using Bayesian information criterion [15]. Analyses found that the combination of IL-8, VEGF, and APOE were ranked number one 410 times (41%), the combination of VEGF and APOE ranked number one 379 times (38%), and all other combinations ranked number one less than 100 times (<10%). The combination of IL-8, VEGF, and APOE achieved a performance almost as good as all 8 biomarkers combined (**Figure 1**). The 3-biomarker assay had an AUC of 0.968 (95% confidence interval [CI], 0.942–0.992), a sensitivity of 90% and specificity of 97% (overall accuracy 93%). The combination of VEGF and APOE had a reduced overall accuracy (89%). The 2-biomarker assay had an AUC of 0.957 (95% confidence interval [CI], 0.927–0.987), a sensitivity of 81% and a specificity of 97%. For comparison, the BTA-Trak commercial assay achieved a sensitivity of 79%, specificity of 83%, and overall accuracy of 81% (**Figure 1**).

## Discussion

Cancer of the urinary bladder is among the five most common malignancies worldwide. At presentation, more than 80% of bladder tumors are non-muscle invasive papillary tumors which have a 5-year survival rate of >90%, however, approximately 70% of patients with these lesions develop tumor recurrence within two years of initial diagnosis. The recurrence phenomenon of non-muscle invasive BCa makes it one of the most prevalent cancers world-wide. Once BCa is detected and treated, patients will routinely get frequent surveillance cystoscopy to monitor for tumor recurrence. If left untreated, initially non-invasive tumors can progress to muscle-invasive tumors which have a significantly reduced 5-year survival rate. Thus, accurate detection of BCa, ideally through non-invasive urine-based analysis, remains an urgent goal.

In order to validate a panel of promising biomarkers discovered using genomics and proteomics approaches [4], [5], we have performed a series of studies using ELISA-based assays [6]–[9]. In this study, we investigated an additional target and combined all data -14 biomarkers in a cohort of 127 subjects- to derive the most accurate diagnostic multi-analyte assays. We also monitored the performance of the commercial BTA-Trak assay in the same cases for comparison, and we quantitated hemoglobin in each case in order to investigate potential correlations between biomarkers and hematuria. Two biomarkers, CCL18 and A1AT, were found to have a relatively high correlation with urinary hemoglobin, raising the possibility that the source of these proteins is, at least in part, serum introduced through bleeding. We will continue to investigate these two biomarkers in independent cohorts, but they were not included in the logistic regression analyses performed to identify optimal diagnostic biomarker combinations. An 8-biomarker panel (IL-8, MMP-9, PAI-1, VEGF, ANG, CA-9, APOE, MMP-10) proved to be the most accurate (overall accuracy of 94%) multi-analyte assay for the detection of BCa in the 127-subject cohort. With multiplex molecular signatures, it is usual that a few of the components will provide much of the predictive power, but additional markers will make the model more robust to errors [4]. By testing all possible combinations via a bootstrap strategy, we revealed that three of the 8 biomarker panel (IL-8, VEGF and APOE) contributed the most information. As a stand-alone assay the 3-biomarker panel achieved an overall accuracy of 93% and maintained both high sensitivity (90%) and specificity (97%). To achieve high accuracy with as few biomarkers as possible is optimal for practical reasons, but it is important that we monitor all of the promising biomarkers further in larger, more diverse cohorts, because different combinations may be more robust in specific conditions, such as the presence of infection, or be more accurate for recurrence versus initial diagnosis.

The components of the 3-biomarker panel (VEGF, IL-8, APOE) have been associated with a number of cancers, including bladder cancer, to varying degrees. In a study of 26 subjects, Crews *et al.* demonstrated that elevated urinary levels of VEGF correlated with levels in excised tissue [17]. In a study of 219 Middle East subjects, urinary levels of VEGF were significantly higher in patients with BCa. In their cohort measuring VEGF outperformed VUC, achieving a diagnostic sensitivity of 76% [18]. Similarly, Bian demonstrated an improved sensitivity of urinary VEGF compared to VUC, achieving 69% vs. 38%, respectively [19]. In another study, patients with elevated urinary VEGF levels had a higher risk of disease recurrence [20]. Previous reports have implicated IL-8 in bladder tumor biology and its use as a potential biomarker of BCa has been investigated in a few studies. IL-8 has been shown to have mitogenic and angiogenic properties, and high levels result in increased tumorigenicity, progression and metastasis in mouse models, reportedly via regulation of nuclear factor kappa-B [21]. Clinical studies have indicated that elevated urinary levels of IL-8 can be associated with the presence of bladder cancer. In a study of 140 subjects, an IL-8 assay achieved a sensitivity of 59%, and a specificity of 90% [22], and in a study of 79 subjects, a sensitivity of 50% and a specificity of 90% was reported [23]. These results are very much in line with our findings when assessing IL-8 as an individual biomarker [7]. Data is scarce on the role of APOE in cancers. Functions include enhancement of lipid transport into cells and mediation of signal transduction upon binding to lipoprotein receptors [24], [25]. APOE has been shown to interact with the transitional epithelial response gene (TERE1), a tumor suppressor gene, in bladder tumor cells, resulting in increased cell turnover and resistance to apoptosis [26], [27]. Recently, Lindén *et al.* reported that APOE was a component of a panel of biomarkers detected by mass spectroscopy, and may be associated with non-muscle invasive BCa [28].

We recognize that our study has several limitations.First as a tertiary care facility, we tend to see more high-grade, high-stage disease, which is reflected in our study cohort. To confirm the robustness of our signature, subsequent studies must assess larger cohorts that include subjects with low-grade, low-stage disease. Second, processed, banked urines were analyzed. Urines were centrifuged and separated into cellular pellet and supernatant prior to storage at −80°C. It is feasible that freshly voided urine samples may provide different results, and it is fresh urine that would be the material used for point-of-care assays. We are currently investigating the performance of selected biomarkers in urines processed via a number of different protocols, including freshly voided urines. Next, the sensitivity of VUC in our cohort of predominantly high-grade (grade 3) disease (33%) was lower than would be expected. This calls into question the known inter-observer variability of interpreting VUC. In subsequent studies, we will utilize two cytopathologists to interpret these results. Furthermore, it is uncertain how the protein composition of the urine supernatant may change during frozen storage. The number of freeze-thaw cycles was kept to 1–2 by dividing the urine supernatant into multiple small aliquots. Lastly, our sample size of 127 is small and the two groups that comprised the 127 subjects were relatively homogeneous, i.e. either active cancer, or control cases with no active cancer, no history of cancer, no urinary tract infection, no urolithiasis, and no gross hematuria. Thus we were not able to assess sensitivity/specificity of our biomarkers among different stages/grades.The specificity of promising biomarkers need to be tested in cohorts that are known to be problematic with other urine-based assays (e.g., hematuria, urinary tract infection, stones and voiding dysfunction).

The identification of robust BCa-associated biomarkers and the establishment of multiplex urine-based assays will have multiple short-term and long-term impacts. Many of the validated biomarkers in this study have not been associated with cancer previously. Analysis of the role of these proteins in tumor cell biology may elucidate mechanisms in tumor initiation or progression, and may reveal novel therapeutic targets. Clinically, accurate BCa assays will have a clear impact on initial diagnostic performance, and on the clinical management of patients post-treatment. If reliable urinary diagnostic biomarker assays can reduce the number of necessary invasive and uncomfortable cystoscopies, improvements in patient compliance and satisfaction will follow, and increased efficiency and cost-savings will benefit the healthcare system. The ultimate goal is to be able to detect BCa in a timely manner such that the patient can expect an improved quality of life and overall outcome.

### Conclusions

Through discovery phase studies that applied advanced profiling techniques to the actual, preferred clinical test analyte, in this case, voided urine, we have identified molecular signatures that can detect BCa with exceptional accuracy. Validation of combinations of these urinary biomarkers using immunochemical assays confirms that multi-analyte tests could significantly improve the non-invasive detection of BCa, and provide a rationale for larger, prospective studies to be undertaken.
